# Polycystic Ovary Syndrome and Gut Microbiota: Phenotype Matters

**DOI:** 10.3390/life13010007

**Published:** 2022-12-20

**Authors:** Larisa Suturina, Natalia Belkova, Ilia Igumnov, Ludmila Lazareva, Irina Danusevich, Iana Nadeliaeva, Leonid Sholokhov, Maria Rashidova, Lilia Belenkaya, Aleksey Belskikh, Eldar Sharifulin, Kseniia Ievleva, Natalia Babaeva, Irina Egorova, Madinabonu Salimova, Mikhail Kuzmin, Daria Tiumentseva, Elizaveta Klimenko, Tuyana Sidorova, Alina Atalyan

**Affiliations:** Federal State Public Institution «Scientific Center for Family Health and Human Reproduction Problems», 16, Timiryazeva Str., 664003 Irkutsk, Russia

**Keywords:** PCOS, phenotype, hyperandrogenism, gut microbiota, alpha diversity indexes, amplicon metasequencing

## Abstract

Abnormalities in gut microbiota diversity are considered important mechanisms in metabolic disorders in polycystic ovarian syndrome (PCOS). However, the data on the association of these disorders with the PCOS phenotype remain controversial. The objectives of this study were to estimate the alpha diversity of the gut microbiota of healthy women and PCOS patients depending on phenotype. The study participants (184 premenopausal women: 63 with PCOS, 121 without PCOS) were recruited during the annual employment assessment in the Irkutsk Region and the Buryat Republic (Russia) in 2016–2019. For PCOS diagnosis, we used the Rotterdam (2003) criteria and definitions of PCOS phenotypes. Five indexes of alpha diversity (ASV, Shannon, Simpson, Chao, and ACE) were estimated for the gut microbiota in all participants using amplicon metasequencing. As a result, two out of five alpha diversity indexes showed a statistical difference between the non-PCOS and PCOS groups. We did not find a significant difference in the alpha diversity of gut microbiota in the subgroups of women with hyperandrogenic PCOS phenotypes vs non-androgenic phenotype D and the group of women with the presence of only one of the PCOS criteria. Nevertheless, “classic” PCOS phenotypes demonstrated the most significant decrease in alpha diversity compared with healthy women without any signs of PCOS.

## 1. Introduction

PCOS is the most common type of hyperandrogenism (HA), associated with reproductive disorders, a high risk of metabolic syndrome, diabetes mellitus, cardiovascular diseases, and psycho-emotional disturbances [1,2]. Previously, it was shown that neuroendocrine regulation and metabolic disorders are related to gut microbiome abnormalities, and convincing evidence has shown that the intestinal microbiomes of sick people differ significantly in the diversity of microorganisms and their potential functionality from the microbiomes of healthy people [3,4,5]. These studies motivate the assumption that human health, to some extent, depends on the taxonomic composition and metabolic functions performed by the intestinal microbiota. In 2012, a hypothesis called DOGMA (dysbiosis of gut microbiota, intestinal microbiota dysbiosis) suggested that intestinal dysbiosis can lead to chronic inflammation, insulin resistance, and androgen hypersecretion, which, in turn, are associated with PCOS [6]. In regard to this hypothesis, the associative relationship between the intestinal microbiota and various pathological conditions and indicators in patients with PCOS is being actively studied (Table S1) [7,8,9,10,11,12,13,14,15,16,17,18,19,20,21,22,23,24,25,26,27,28,29,30,31,32,33]. 

Microbial biocenoses formed in different biotopes of the human body consist of a number of species and their quantitative composition and bacterial diversity. Various bioinformatics tools have been developed to compare bacterial diversity in different biotopes [34,35]. The Shannon and Simpson diversity indexes are used to estimate bacterial diversity based on operational taxonomic units (OTUs). The Shannon index describes the species richness and species uniformity with an emphasis on species richness. The Simpson index provides an estimate of species richness and species uniformity with an emphasis on species uniformity. While the Abundance-based Coverage Estimator (ACE) and Chao1 indexes are used to estimate species richness, they determine expected OTUs based on the OTUs found [36,37]. The Chao index gives more weight to species with low abundance, so this index is especially useful for datasets biased towards low-abundance species [38]. The ACE index divides the observed frequencies into abundant and rare groups, considering only the information about the presence or absence of numerous species because they will be found anyway. However, the estimate of the number of missing species is entirely based on rare species, so precise frequencies are required for rare species [36,37]. Currently, there is a lack of studies based on the estimation of the spectrum of diversity indexes; the role of gut microbiota diversity in PCOS has not been finally established, and the available results are contradictory. The objectives of this study were to estimate the alpha diversity of the gut microbiomes of healthy women and PCOS patients depending on phenotype.

## 2. Materials and Methods

### 2.1. Study Participants

All study participants (*n* = 184) were recruited during the multicenter, institution-based, cross-sectional Eastern Siberia PCOS Epidemiology & Phenotype (ESPEP) study performed in the Irkutsk Region and the Buryat Republic (Russia) in 2016–2019 (ClinicalTrials.gov ID: NCT05194384). The study was conducted in accordance with the Declaration of Helsinki 1964, with subsequent changes, and approved by the Local Ethics Committee of the Scientific Center for Family Health and Human Reproduction Problems (protocol number 2.1, date of approval 24 February 2016). Written informed consent was obtained from all participants.

Inclusion criteria were as follows: premenopausal women, aged 18 to 44 yrs, who underwent an obligatory early medical employment assessment, provided a signed informed consent form, were available during the study, and complied with all study procedures.

Exclusion criteria were: current pregnancy or lactation; history of hysterectomy, bilateral oophorectomy, endometrial ablation, or uterine artery embolization; current or previous (within 3 months) hormonal medications or insulin sensitizers intake; intake of antibiotics within one month of recruitment; anything that would place the individual at increased risk or preclude the individual’s full compliance with or completion of the study; and unwillingness to participate or difficulty understanding the consent processes or the study objectives and requirements.

For PCOS diagnosis, we used Rotterdam (2003) criteria: a presence of any two of three criteria—hyperandrogenism, oligo/anovulation, and polycystic ovarian morphology with the absence of conditions with similar symptoms (hyperprolactinemia, hypothyroidism, 21-hydroxylase-deficient non-classic congenital adrenal hyperplasia (NC-CAH), premature ovarian failure) [39]. PCOS phenotypes were defined based on the combination of clinical and biochemical PCOS features as follows: phenotype A—clinical and/or biochemical hyperandrogenism (HA) and oligo/anovulation (OA)/menstrual dysfunction (MD) and polycystic ovarian morphology (PCOM); B-HA and OA/MD; C-HA and PCOM; and D-OA/MD and PCOM [39].

All included women with PCOS and without PCOS were divided into 4 subgroups. The control subgroup (1a, *n* = 19) included women without any signs of PCOS, with regular 21–35-day menstrual cycles; a modified Ferriman–Gallwey (mF-G) score < 3, without alopecia or acne; and with an ovarian volume by pelvic ultrasound < 10 cm^3^ and antral follicle count (AFC) less than 12. Subjects with a history of chronic disease; BMI < 18 or ≥30 kg/m^2^; elevated blood pressure; or abnormal androgens, fasting glucose, prolactin, FSH, TSH, and 17-hydroxyprogesterone levels were not included in the 1a (control) group. Women from the “Grey zone” subgroup (1b, *n* = 102) met one of the three possible PCOS criteria: hyperandrogenism, oligo/anovulation, or PCOM. The 2a subgroup (*n* = 41) included patients with “hyperandrogenic” PCOS phenotypes (A, B, and C), and the 2b subgroup (*n* = 22) consisted of patients with “non-androgenic” phenotype D.

### 2.2. Methods

Subject evaluations included questionnaires, anthropometry, measurement of vital signs, gynecological examination, modified Ferriman–Gallwey (mF-G) scoring [40], pelvic ultrasound, and blood sampling in the morning after an overnight fast. Patients were examined during annual check-ups, and blood samples were taken both in the first and second phases of the menstrual cycle. A body mass index (BMI) was calculated with the following: weight (kg)/height (m^2^). Pelvic ultrasound (U/S) was performed using Mindray M7 (MINDRAY, China), a transvaginal probe (5.0–8.0 MHz), or transabdominal probe (2.5–5.0 MHz). Ovarian volume was determined by the following formula: length × width × height × 0.523.

Serum samples were analyzed for total testosterone (TT) using LC-MS/MS. DHEAS, sex-hormone-binding globulin (SHBG), prolactin, LH, FSH, TSH, AMH, and 17-OHPwere assessed by ELISA. The free androgen index (FAI) was calculated by: [TT/SHBG] × 100.

Sampling for high-throughput sequencing was performed according to standard operating procedures (SOP: IHMS_SOP03 V2, IHMS_SOP06 V2) developed during the implementation of the International Human Microbiome Standards (IHMS) project of the international consortium (http://www.human-microbiome.org/index.php#SOPS, accessed on 3 March 2016). On the eve of admission, patients received a home fecal collection kit, which included a sterile fecal jar, refrigerant, and instructions. The patients transported the biomaterial in refrigerant to the laboratory within 2 h after defecation. The feces were immediately divided into aliquots for storage at −80 °C until further processing. 

Genomic DNA was isolated from feces using the Quick-DNA Fecal/Soil Microbe Kit (Zymo Research, Irvine, CA, USA).

Library preparation and sequencing were carried out in accordance with the manufacturer’s recommendations: amplified fragments were indexed using the Nextera XT Index kit v2 (set A-D), and individual libraries were mixed in equimolar amounts and sequenced on an Illumina MiSeq instrument (Illumina, San Diego, CA, USA) using a MiSeq^®^ Reagent Kit v3 (600 cycle) with double-sided reading (2 × 300 N).

Sequencing of the V1-V3 amplicons of the variable regions of the 16S rRNA gene was performed using the equipment of the Core Centrum ‘Genomic Technologies, Proteomics and Cell Biology’ in ARRIAM, and the primary data were deposited in the international database NCBI SRA (data: PRJNA899143).

Amplicon libraries of 16S rDNA were processed using the QIIME2 bioinformatics pipeline to conduct a comparative metagenomic study [41]. ASVs were generated using the DADA2 algorithm, which allows detection, correction, and filtering of amplicon errors and chimeric sequences. The resulting representative sequences were used to determine their taxonomic classification using the sklearn-based Naive Bayes classifier trained on the SILVA v138 with 99% 16S full-length database. Diversity analysis was performed to estimate alpha diversity using the “diversity” plugin, and species richness difference analysis was performed using the following indexes: Chao1, Shannon, Simpson, and ACE.

Statistical analysis: Sample size calculations for the total population were based on the following formula: *n* = [(Z1 − α )^2^ (*p*(1 − P)/D2)] [42], where *n* = individual sample size, Z1 − α = 1.96 (when α = 0.05), *p* = assumed PCOS prevalence according to the previously published data [1,2], and D = absolute error. The data were collected using Research Electronic Data Capture (REDCap) [43]. Outliers were identified during the Exploratory Data Analysis [44,45] using the box-plot and 3σ methods. Managing missing data: In our research dataset, there were two types of missing data: missing completely at random (MCAR) and missing at random (MAR). We recorded all missing values with labels of “N/A” to make them consistent throughout our dataset. Pairwise deletion was used when the dataset was analyzed.

To estimate the assumption of the normal distribution of our datasets, we performed a formal statistical test—the Shapiro–Wilk test. Data are presented as means ± standard deviation if normally distributed and as median and interquartile range in case of a skewed distribution. Chi-square (χ^2^) was used for frequency data. We used a Student’s t-test to compare the mean values of the data with an independent sample, which followed a normal distribution, or a Mann–Whitney U test to compare the ratio between two groups in another case. For comparing means in a situation where there were more than two groups, we performed one-way analysis of variance (ANOVA) or a non-parametric alternative—the Kruskal–Wallis rank-sum test with the post hoc tests including the Bonferroni correction: Tukey multiple pairwise comparisons or a modified version of the U test as a post hoc test in the case of a non-parametric test. The Spearman rank-order correlations were calculated for the alpha diversity indexes and hormonal parameters for all women.

All data were analyzed using R 3.6.3 (a free software environment for statistical computing and graphics: https://www.r-project.org/, accessed on 19 September 2022) and packages rstatix 0.7.0., ggplot2, and catdap1.3.5. 

## 3. Results

### 3.1. The Study Participants’ Characteristics

We present the sociodemographic characteristics of the women included in the study in Table S2. A comparison of the physical examination data demonstrates significantly higher mFG scores in patients with PCOS vs those without PCOS (Table 1).

As shown in this table, the patients with PCOS had ultrasound signs of polycystic ovarian morphology: an increased ovarian volume and antral follicle count per ovary.

Anthropometric data, vital signs, and U/S parameters of the study participants by subgroups are presented in Table 2.

The main hormonal characteristics of non-PCOS and PCOS women, overall and by phenotype, presented in Table 3 and Table 4, demonstrate the higher values of LH, AMH, and androgens in women with PCOS, especially in those who have hyperandrogenic PCOS phenotypes.

### 3.2. Differences in Alpha Diversity of Gut Microbiota According to Study Group and Subgroups

**Figure 1:**
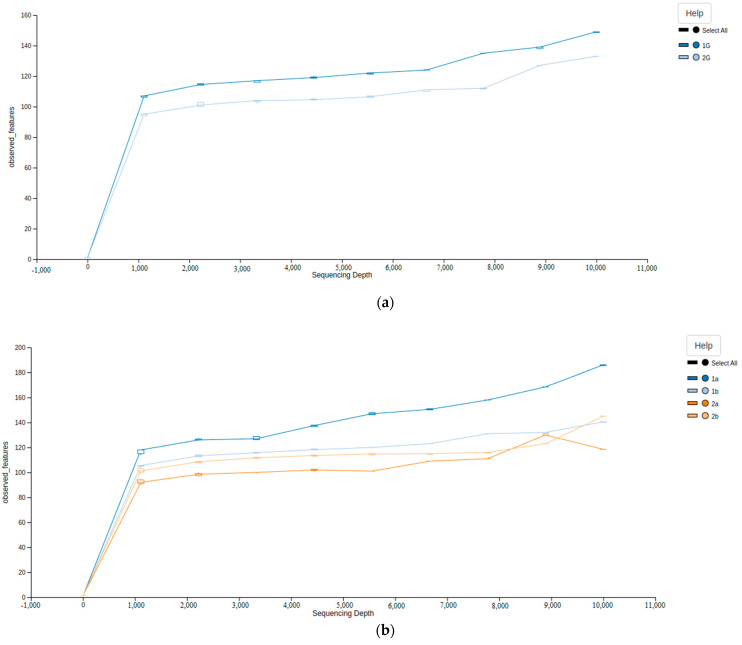
Alpha diversity rarefaction curves based on the number of observed ASVs. (**a**) Non-PCOS (**1**) and PCOS (**2**) groups; (**b**) subgroups: Control (**1a**), “Grey zone” (**1b**), Phenotype A, B, C (**2a**), and Phenotype D (**2b**). All samples were rarefied to 10,000 reads and then clustered into ASV. Mean and SD are plotted.

The sequencing of the V1–V3 16S microbial rDNA generated a dataset consisting of 4,460,863 total reads. After filtering, denoising, chimeric read removal, and merging of the reads, there were 2,232,329 high-quality 16S rDNA gene sequences with 758,882 reads for non-PCOS women and 1,473,447 reads for women with PCOS, with an average of 12,132 sequences per sample (minimum, 2952; maximum, 33,855) (Table S3). The rarefaction curves were used to establish whether the sequencing quantity was sufficient and estimate the species richness (Figure 1). The curve seemingly flattened, indicating that the sequencing depth was sufficient to reflect the species diversity of the samples.

**Figure 2:**
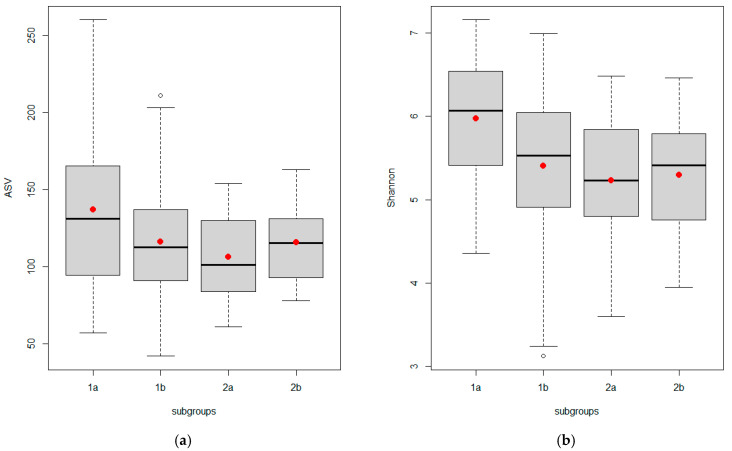

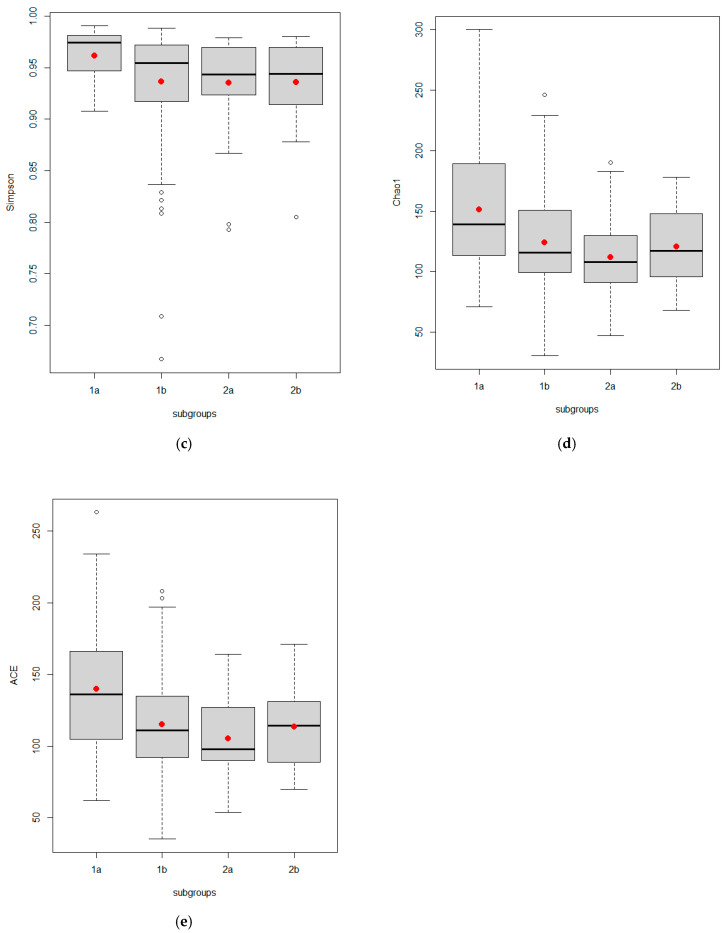
Indexes of alpha diversity of the gut microbial communities from Control (*n* = 19) and “Grey zone” (*n* = 102) of non-PCOS group and phenotypes A, B, C (*n* = 41) and phenotype D (*n* = 22) of PCOS group: (**a**) ASV, (**b**) Shannon, (**c**) Simpson, (**d**) Chao, and (**e**) ACE. The box represents the interquartile range, which contains the median, and the whiskers indicate the 75th and 25th percentiles.

Indexes of alpha diversity (ASV, Shannon, Simpson, Chao, and ACE) were estimated for the gut microbiota in the two groups (Table 5) and four subgroups (Figure 2, Table 6) of the women. As a result, two out of five alpha diversity indexes showed a statistical difference between the non-PCOS and PCOS groups (Shannon; *p* = 0.039 and Chao; *p* = 0.048, respectively) (Table 5). We did not find a significant difference in the alpha diversity of the gut microbiota in the subgroups of women with hyperandrogenic PCOS phenotypes (subgroup 2a) vs non-androgenic phenotype D (subgroup 2b) and vs the subgroup of women with the presence of only one of the PCOS criteria (1b or “Grey zone”) (Figure 2, Table 6).

Nevertheless, “classic” hyperandrogenic PCOS phenotypes (subgroup 2a) demonstrated the most significant decrease in alpha diversity, estimated by five indexes, compared with healthy women without any signs of PCOS (subgroup 1a) (Figure 2, Table 6). Two out of five alpha diversity indexes (Shannon and Simpson) showed a statistical difference between the Control (1a) and “Grey Zone” (1b) subgroups of women without PCOS and between controls and PCOS phenotype D (2b) subgroup.

Investigation of the correlations of bacterial richness and diversity with hormonal parameters for all women revealed that such characteristics as total testosterone, DHEAS, SHBG, and LH did not significantly correlate with any of the alpha diversity indexes, whereas a negative correlation was revealed for FAI with only Chao richness (r = −0.15, *p* = 0.041), and FSH with all indexes except Simpson (ASV: r = −0.15, *p* = 0.044; Shannon: r = −0.15, *p* = 0.040; Chao: r = −0.18, *p* = 0.013; ACE: r = −0.16, *p* = 0.031).

## 4. Discussion

In recent decades, microbial communities from various human biotopes have been actively studied and it has been shown that they differ under normal and pathological conditions. The gut microbiota is a group of bacteria that stably exist in association with the intestinal epithelium. These microorganisms represent a stable community within which they realize interrelated and complementary functions [46]. Ecological principles underlie both host–microbe interactions and the specific functions of gut microorganisms. The microorganism’s diversity, metabolic “flexibility”, and functional redundancy ensure the stability and sustainability of any microbial community.

The diversity of microorganisms in a microbiocenosis can be assessed using a set of indicators that consider not only the number of different taxa present in the community (richness) but also their uniformity and evenness [47].

We evaluated the five most commonly used metrics to characterize alpha diversity—ASV, Shannon, Simpson, ACE, and Chao1. The values of all indicators significantly differed only between the control and the “classic“ hyperandrogenic phenotypes of PCOS (the subgroup consisting of phenotypes A, B, and C). Notably, in previous studies, the authors did not always evaluate all of these main indexes of alpha diversity (Table S1). Studies that contain a shotgun sequencing analysis do not explore the information on alpha diversity indexes [14]. The most commonly used index is the Shannon index, and the taxonomic complexity, richness, and evenness of the gut microbiota could be best analyzed using this indicator. Most often, the authors point to significantly lower values of this indicator in patients with PCOS (Table S1) compared with controls. As usual, data that demonstrate the absence of a difference between patients with PCOS and the control group were obtained from the research that studied the association of gut microbiota with obesity, and the patients with and without PCOS were not stratified by this important comorbidity [8,11,15,20]. Among the publications currently available for analysis, we found four studies in which the authors note higher values of all indexes analyzed or at least one index in patients with PCOS compared with the control group [9,20,24,29]. In all these studies, the authors revealed the relationships of the clinical parameters with the species composition of the gut microbiota. Nevertheless, in general, they did not describe the abnormalities of the alpha diversity of the gut microbiota.

The associative relationships between the gut microbiome and clinical parameters have been presented in several studies [8,13,14,18,20,21,23,24,25,28,30]. However, the authors characterized the associations of only certain groups of bacteria that showed a greater or lesser representation in the gut microbiome. We have found only a few references where the associations of alpha diversity indexes with clinical, hormonal, or metabolic parameters were investigated [10,26,33]. When using the correlation analysis of alpha diversity indexes in PCOS and non-PCOS women, the authors reported no statistical significance. The lack of differences in the diversity indexes could be explained by the absence of PCOS phenotyping. Importantly, in our study, the most significant difference regarding the diversity was demonstrated between the control group and “classic” phenotypes of PCOS.

Recently, Lüll with co-authors carried out a correlation analysis of alpha diversity indexes with clinical, hormonal, and metabolic parameters for the participants of the population-based study of gut microbiome associations with PCOS [33]. They found a negative correlation for the Shannon index with BMI, fasting insulin, and FAI, and a positive correlation with SHBG, Matsuda index, and Disposition index. Obviously, in this study, age and BMI may have significantly influenced the association of alpha diversity and PCOS.

Some data were obtained from the study of the potential relationship between gut microbiota and PCOS in a well-characterized homogenous group of lean women with PCOS compared with healthy women. In this study, such characteristics as age, BMI, lipids, clinical or biochemical androgen excess, and insulin resistance did not significantly impact Shannon diversity or Chao richness [26]. The authors noted that Chao richness showed a borderline negative correlation with androstenedione and an insignificant negative correlation with testosterone. To the contrary, Torres and co-authors have shown negative correlations of serum total testosterone level and hirsutism with observed ASVs and Faith PD [10].

Therefore, these studies presented controversial results, and we believe that our data increase the body of evidence regarding the associations of hyperandrogenic PCOS phenotypes with the most significant decrease in gut microbiota diversity.

The main strength of our study is the fact that all study participants were recruited from a representative, medically unbiased population. All study participants were well phenotyped for PCOS, and we used a highly efficient method (LC-MS/MS) to measure testosterone levels. To assess the alpha diversity of gut microbial communities, we explored a panel of indexes and described diversity in a more comprehensive manner than the previously published data.

A limitation of our study is the relatively small sample size of the control subgroup compared with the size of the other subgroups, but it is comparable to the other studies (Table S1). We have not yet conducted a search on the composition of the gut microbiota, but this will be the result of the next separate study.

## 5. Conclusions

In this study, we demonstrated that even alpha diversity indexes describing the characteristics of the microbiota of each individual patient provide information that makes it possible to distinguish the microbiome of a healthy person from a patient with a “classic” PCOS phenotype. Notably, in addition to the gut microbiome, other biotopes of patients with PCOS are being actively studied, for example, the oral cavity [48,49], vaginal microbiome [50,51,52], blood bacteria spectrum [53], and gut virome [54]. Undoubtedly, for a comprehensive characterization of the microbiome in PCOS, it is necessary to consider a complex of factors of bacterial origin.

## Figures and Tables

**Table 1:** Anthropometry, vital signs, and U/S parameters of the study participants with and without PCOS.

Parameters	Non-PCOS *n* = 121	PCOS *n* = 63	*p*-Value
Age, years, Mean ± SD	35.11 ± 5.71	29.48 ± 5.21	<0.001 ^#^
Height, cm, Mean ± SD	163.71 ± 6.00	164.93 ± 6.57	0.07 ^#^
Weight, kg, Mean ± SD	71.95 ± 16.11	71.29 ± 14.73	0.98 ^#^
BMI, kg/m^2^, Mean ± SD	26.83 ± 5.83	26.24 ± 5.24	0.65 ^#^
mFG score, M ± SD Me [25% Quartile; 75% Quartile]	1.27 ± 1.91 0.00 [0.00; 2.00]	3.67 ± 3.61 3.00 [0.00; 6.00]	<0.001 *
Waist circumference, cm Mean ± SD	80.36 ± 12.91	79.29 ± 13.19	0.48 ^#^
Hip circumference, cm, Mean ± SD	101.62 ± 10.62	100.83 ± 9.44	0.73 ^#^
Systolic blood pressure, mm Hg, Mean ± SD	126.02 ± 14.09	121.54 ± 12.37	0.03 ^#^
Diastolic blood pressure, mm Hg Mean ± SD	81.07 ± 9.39	78.86 ± 9.49	0.12 ^#^
Pelvic U/S	M ± SD Me [25% Quartile; 75% Quartile]		
AFC, right ovary	7.88 ± 3.75 7.00 [5.00; 9.00]	12.27 ± 3.87 12.00 [10.00; 14.00]	<0.001 *
AFC, left ovary	7.16 ± 3.22 6.00 [5.00; 9.00]	11.50 ± 3.53 12.00 [10.00; 13.00]	<0.001 *
Volume, right ovary, cm^3^	13.87 ± 52.08 6.92 [5.51; 9.06]	13.71 ± 9.12 11.68 [10.01; 13.41]	<0.001 *
Volume, left ovary, cm^3^	8.38 ± 8.95 6.44 [5.05; 8.69]	10.79 ± 4.64 9.37 [7.64; 12.91]	<0.001 *

* Mann–Whitney U Test. #—Student’s test. Abbreviations: AFC—antral follicle count, BMI—body mass index, mFG—modified Ferriman–Gallwey score for hirsutism, U/S—ultrasound.

**Table 2:** Anthropometry, vital signs, and U/S parameters of the study participants by subgroups.

Parameter	Non-PCOS *n* = 121		PCOS *n* = 63		*p*-Value
	Control (1a) *n* = 19	«Grey Zone» (1b) *N* = 102	Phenotypes A, B, C (2a) *n* = 41	Phenotype D (2b) *n* = 22	
Age, years, Mean ± SD	34.3 ± 4.3	35.3 ± 5.9	29.8 ± 5.5	28.9 ± 4.7	<0.001 * p_1a-1b_ = 1.00 *p*_1a-2a_ = 0.03 *p*_1a-2b_ = 0.02 *p*_1b-2a_ < 0.001 *p*_1b-2b_ < 0.001 *p*_2a-2b_ = 1.00
Height, cm, Mean ± SD	162.91 ± 5.9	163.9 ± 6.0	164.6 ± 7.3	165.7 ± 5.2	0.53 *
Weight, kg, Mean ± SD	65.7 ± 12.4	73.1 ± 16.5	73.3 ± 16.4	67.7 ± 10.2	0.13 *
BMI, kg/m^2^, Mean ± SD	24.6 ± 3.5	27.3 ± 6.1	27.1 ± 5.6	24.7 ± 4.1	0.08 *
mFG score, M ± SD Me [25% Quartile; 75% Quartile]	0.42 ± 0.69 0.00 [0.00; 1.00]	1.43 ± 2.02 0.50 [0.00; 2.00]	5.05 ± 3.67 5.00 [2.00; 7.00]	1.09 ± 1.48 0.00 [0.00; 2.00]	<0.001 ^#^ *p*_1a-1b_ = 0.52 *p*_1a-2a_ < 0.001 *p*_1a-2b_ = 1.00 *p*_1b-2a_ < 0.001 *p*_1b-2b_ = 1.00 *p*_2a-2b_ < 0.001
Waist circumference, cm, Mean ± SD	75.9 ± 8.9	81.2 ± 13.4	81.8 ± 13.6	74.6 ± 11.4	0.07 *
Hip circumference, cm, Mean ± SD	98.1 ± 8.3	102.3 ± 10.9	102.4 ± 10.2	97.9±7.1	0.14 *
Systolic blood pressure, mm Hg, Mean ± SD	117.3 ± 11.7	127.7 ± 13.9	123.8 ± 12.6	117.4±11.2	<0.001 * p_1a-1b_= 0.008 *p*_1a-2a_ = 0.47 *p*_1a-2b_ = 1.00 *p*_1b-2a _ = 0.52 *p*_1b-2b _ = 0.005 *p*_2a-2b_ = 0.41
Diastolic blood pressure, mm Hg, Mean ± SD	75.4 ± 7.3	82.1 ± 9.4	80.3 ± 9.3	76.1±9.4	0.002 * p_1a-1b_ = 0.015 *p*_1a-2a_ = 0.31 *p*_1a-2b_ = 1.00 *p*_1b-2a _ = 1.00 *p*_1b-2b_ = 0.02 *p*_2a-2b_ = 0.48
Pelvic U/S	M ± SD Me [25% Quartile; 75% Quartile]				
AFC, right ovary	6.63 ± 3.15 6.00 [5.00; 8.00]	8.11 ± 3.82 7.00 [5.00; 10.00]	12.02 ± 3.54 12.00 [11.00; 14.00]	12.73 ± 4.47 12.00 [10.00; 13.00]	<0.001 ^#^ *p*_1 a-1b_ = 0.26 *p*_1a-2a_ < 0.001 *p*_1a-2b_ < 0.001 *p*_1b-2a_ < 0.001 *p*_1b-2b_ < 0.001 *p*_2a-2b_ = 1.00
AFC, left ovary	6.47 ± 2.46 6.00 [5.00; 8.00]	7.29 ± 3.34 6.00 [5.00; 9.50]	11.28 ± 3.95 12.00 [8.50; 13.00]	11.91 ± 2.64 0.00 [0.00; 2.00]	<0.001 ^#^ *p*_1a-1b_ = 1.00 *p*_1a-2a_ = 0.006 *p*_1a-2b_ < 0.001 *p*_1b-2a_ = 0.009 *p*_1b-2b_ < 0.001 *p*_2a-2b_ = 0.18
Volume, right ovary, cm^3^	6.40 ± 1.71 6.12 [4.85; 7.46]	15.26 ± 56.65 7.40 [5.52; 9.49]	12.56 ± 6.70 11.60 [9.54; 13.27]	15.86 ± 12.36 11.81 [10.24; 13.41]	<0.001 ^#^ *p*_1a-1b_ = 0.52 *p*_1a-2a_ < 0.001 *p*_1a-2b_ < 0.001 *p*_1b-2a_ < 0.001 *p*_1b-2b_ < 0.001 *p*_2a-2b_ = 1.00
Volume, left ovary, cm^3^	6.20 ± 2.00 6.44 [4.61; 7.18]	8.80 ± 9.68 6.41 [5.17; 8.87]	9.54 ± 3.91 8.79 [7.07; 12.60]	13.06 ± 5.07 11.76 [8.84; 16.95]	<0.001 ^#^ *p*_1 a-1b_=1.000 *p*_1a-2a_ < 0.001 *p*_1a-2b_ < 0.001 *p*_1b-2a_ < 0.001 *p*_1b-2b_ < 0.001 *p*_2a-2b_ = 1.000

* Kruskal–Wallis ANOVA by Ranks Multiple Comparisons two-tailed method (post hoc); ^#^ ANOVA Multiple Comparisons Tukey method (post hoc). Abbreviations: AFC—antral follicle count, BMI—body mass index, mFG—modified Ferriman–Gallwey score for hirsutism, U/S—ultrasound.

**Table 3:** Hormonal characteristics of study participants with and without PCOS.

Parameter	Non-PCOS *n* = 121	PCOS *n* = 63	*p*-Value *
M ± SD Me [25%Quartile; 75% Quartile]			
Prolactin, mIU/mL	358 ± 249 292 [206; 436]	314 ± 145 278 [221; 383]	0.61
TSH, mIU/mL	1.96 ± 1.795222 1.50 [1.10; 2.10]	1.57 ± 0.71 1.60 [1.00; 1.90]	0.48
LH, mIU/mL	7.10 ± 7.74 5.25 [3.00; 7.65]	10.82 ± 8.44 8.60 [5.40; 14.00]	<0.001
*follicule phase, mIU/mL*	8.28 ± 8.21 5.90 [4.90; 9.10]	9.42 ± 4.94 7.45 [5.60; 13.20]	0.09
*luteal phase, mIU/mL*	6.29 ± 7.09 5.00 [2.50; 7.40]	10.61 ± 9.10 8.45 [4.85; 13.50]	0.000
FSH, mIU/mL	6.01 ± 5.93 5.25 [3.75;6.55]	5.44 ± 1.70 5.40 [3.90; 6.40]	0.46
*follicule phase, mIU/mL*	7.09 ± 3.61 6.20 [5.45; 7.00]	5.96 ± 1.02 5.85 [5.60; 6.80]	0.521
*luteal phase, mIU/mL*	4.60 ± 4.60 4.10 [2.90; 5.70]	5.06 ± 1.80 4.95 [3.55;6.15]	0.122
AMH, ng/mL	3.13 ± 3.52 2.20 [0.80; 4.10]	7.71 ± 6.10 5.60 [3.10; 8.80]	<0.001
17OHP, nmol/L	2.57 ± 1.65 2.20 [1.40; 4.20]	4.31 ± 1.33 4.60 [3.70; 5.00]	0.005
TT, ng/dL	29.2 ± 23.8 26.0 [17.1; 36.5]	46.3 ± 26.1 43.2 [27.5; 56.2]	<0.001
*follicule phase, ng/dL*	32.0 ± 32.3 25.7 [14.7; 37.1]	41.3 ± 19.3 47.5 [25.5; 55.6]	0.024
*luteal phase, ng/dL*	28.6 ± 16.1 27.4 [18.8; 36.6]	50.2 ± 30.2 38.5 [28.6; 63.2]	0.000
SHBG, nmol/L	80.4 ± 50.1 71.0 [45.9; 103.9]	66.5 ± 52.1 45.6 [33.0; 89.5]	0.008
FAI	1.96 ± 3.73 1.23 [0.68; 2.07]	3.55 ± 3.01 2.68 [1.39; 4.65]	<0.001
*follicule phase*	2.33 ± 5.40 1.37 [0.80; 2.07]	2.54 ± 1.76 2.01 [1.30; 3.22]	0.029
*luteal phase*	1.77 ± 2.07 1.09 [0.66; 2.05]	3.80 ± 3.04 3.22 [1.44; 5.06]	0.000
DHEAS, μg/dL	168 ± 72.8 161 [116; 218]	232 ± 114 200 [139; 319]	<0.001
*follicule phase, μg/dL*	176 ± 79.0 158 [114; 226]	234 ± 107 218 [134; 311]	0.046
*luteal phase, μg/dL*	165 ± 66.5 166 [119; 215]	225 ± 116 194 [140; 294]	0.016

* Mann–Whitney U Test. Abbreviations: TSH—thyroid-stimulating hormone, LH—luteinizing hormone, FSH—follicle-stimulating hormone, AMH—anti-Mullerian hormone, 17OHP—17 hydroxyprogesterone, TT—total testosterone, SHBG—sex-hormone-binding globulin, FAI—free androgen index, DHEAS—dehydroepiandrosterone sulfate.

**Table 4:** Hormonal characteristics of study participants by subgroups.

Parameter	Non-PCOS *n* = 121		PCOS *n* = 63		*p*-Value *^,#^
	Control (1a) *n* = 19	«Grey Zone» (1b) *n* = 102	Phenotypes A, B, C (2a) *n* = 41	Phenotype D (2b) *n* = 22	
	M ± SD Me [25% Quartile; 75% Quartile]				
Prolactin, mIU/mL	295 ± 131 248 [185; 436]	370 ± 264 298 [217; 436]	316 ± 144 284 [232; 384]	309 ± 151 271 [199; 352]	0.70
TSH, mIU/mL	1.55 ± 0.70 1.60 [0.90; 1.90]	2.03 ± 1.93 1.50 [1.10; 2.10]	1.64 ± 0.81 1.60 [1.00; 2.00]	1.44 ± 0.45 1.45 [1.10; 0.80]	0.75
LH, mIU/mL	6.55 ± 5.24 5.80 [3.90; 7.40]	7.20 ± 8.14 5.20 [2.90; 7.90]	12.0 ± 9.63 8.30 [5.90; 14.50]	8.59 ± 5.05 8.90 [4.20; 11.80]	<0.001 *p*_1a-1b_ = 1.00 *p*_1a-2a_ = 0.04 *p*_1a-2b_ = 1.00 *p*_1b-2a_ < 0.001 *p*_1b-2b_ = 0.29 *p*_2a-2b_ = 1.00
FSH, mIU/mL	5.74 ± 1.85 6.10 [4.00; 6.90]	6.06 ± 6.42 5.00 [3.70; 6.40]	5.81 ± 1.89 5.90 [4.90; 7.20]	4.75 ± 0.98 5.00 [3.70; 5.40]	0.05
AMH, ng/mL	2.79 ± 2.06 2.00 [1.20; 4.60]	3.20 ± 3.75 2.20 [0.70; 4.10]	6.63 ± 5.48 4.70 [2.50; 8.70]	9.72 ± 6.79 7.20 [5.20; 17.20]	<0.001 *p*_1a-1b_ = 1.000 *p*_1a-2a_ = 0.02 *p*_1a-2b_ < 0.001 *p*_1b-2a_ < 0.001 *p*_1b-2b_ < 0.001 *p*_2a-2b_ = 0.39
17OHP, nmol/L	1.95 ± 1.54 2.00 [0.70; 2.80]	2.83 ± 1.67 2.30 [1.50; 4.30]	4.40 ± 0.70 4.40 [3.70; 5.00]	4.20 ± 2.01 4.60 [3.00; 5.40]	0.035 *p*_1a-1b_ = 1.00 *p*_1a-2a_ = 0.08 *p*_1a-2b_ = 0.25 *p*_1b-2a_ = 0.24 *p*_1b-2b_ = 0.65 *p*_2a-2b_ = 1.00
TT, ng/dL	23.5 ± 11.8 25.5 [14.9; 30.1]	30.3 ± 25.3 27.4 [17.6; 37.3]	53.3 ± 27.9 47.7 [31.8; 66.7]	33.3 ± 15.8 30.8 [21.1; 39.6]	<0.001 *p*_1a-1b_ = 1.00 *p*_1a-2a_ < 0.001 *p*_1a-2b_ = 0.50 *p*_1b-2a_ < 0.001 *p*_1b-2b_ = 1.00 *p*_2a-2b_ = 0.05
SHBG, nmol/L	89.0±46.9 68.7 [57.2; 114.7]	78.7 ± 50.8 71.2 [43.6; 102.9]	61.2 ± 45.4 42.0 [32.9; 76.3]	76.3 ± 62.5 66.9 [37.6; 94.3]	0.018 *p*_1a-1b_ = 1.00 *p*_1a-2a_ = 0.03 *p*_1a-2b_ = 1.00 *p*_1b-2a_ = 0.05 *p*_1b-2b_ = 1.00 *p*_2a-2b_ = 1.00
FAI	1.10 ± 0.81 0.97 [0.49; 1.39]	2.12 ± 4.03 1.31 [0.80; 2.10]	4.33 ± 3.35 3.29 [2.06; 5.39]	2.08 ± 1.36 1.67 [0.95; 3.23]	<0.001 *p*_1a-1b_ = 0.54 *p*_1a-2a_ < 0.001 *p*_1a-2b_ = 0.12 *p*_1b-2a_ < 0.001 *p*_1b-2b_ = 1.00 *p*_2a-2b_ = 0.03
DHEAS, μg/dL	173 ± 65.9 186 [118; 215]	167 ± 73.6 153 [114; 219]	256 ± 125 236 [141; 334]	189 ± 75 189 [130; 221]	0.001 *p*_1a-1b_ = 1.00 *p*_1a-2a_ = 0.15 *p*_1a-2b_ = 1.00 *p*_1b-2a_ < 0.001 *p*_1b-2b_ = 1.00 *p*_2a-2b_ = 0.55

* Kruskal–Wallis ANOVA by Ranks; ^#^ Multiple Comparisons two-tailed method (post hoc). Abbreviations: TSH—thyroid-stimulating hormone, LH—luteinizing hormone, FSH—follicle-stimulating hormone, AMH—anti-Mullerian hormone, 17OHP—17 hydroxyprogesterone, TT—total testosterone, SHBG—sex-hormone-binding globulin, FAI—free androgen index, DHEAS—dehydroepiandrosterone sulfate.

**Table 5:** Alpha diversity indexes calculated for two main groups of PCOS and non-PCOS patients with a median relative abundance.

Indexes	Non-PCOS *n* = 121 (1)	PCOS *n* = 63 (2)	P_1–2_ *
ASV	114 (91; 138) **	106 (87; 131)	0.163
Shannon	5.57 (4.93; 6.07)	5.28 (4.77; 5.84)	0.039
Simpson	0.96 (0.92; 0.97)	0.94 (0.92; 0.97)	0.088
Chao	119 (100; 157)	109 (92; 132)	0.048
ACE	113 (93; 145)	103 (89; 127)	0.088

* *p*-values are significant at *p* < 0.05; ** data are expressed as median (25% Quartile; 75% Quartile). Abbreviations: PCOS—polycystic ovary syndrome; ASV—amplicon sequence variant, ACE—abundance-based coverage estimator.

**Table 6:** Alpha diversity indexes calculated for subgroups of patients with a median relative abundance.

Indexes	Non-PCOS *n* = 121		PCOS *n* = 63		*p*-Value *					
	Control (1a) *n* = 19	“Grey Zone” (1b) *n* = 102	Phenotypes A, B, C (2a) *n* = 41	Phenotype D (2b) *n* = 22	1a–1b	2a–2b	1a–2a	1a–2b	1b–2a	1b–2b
ASV	131 ** [85; 166]	113 [91; 137]	101 [84; 130]	116 [93; 131]	0.140	0.108	0.045	0.248	0.127	0.823
Shannon	5.96 [5.36; 6.54]	5.53 [4.91; 6.04]	5.23 [4.80; 5.84]	5.41 [4.76; 5.79]	0.016	0.880	0.003	0.014	0.203	0.357
Simpson	0.97 [0.94; 0.98]	0.95 [0.92; 0.97]	0.94 [0.92;0.97]	0.94 [0.91; 0.97]	0.017	0.971	0.006	0.017	0.351	0.472
Chao	139 [111; 192]	116 [99; 151]	108 [91; 130]	117 [96; 148]	0.075	0.285	0.010	0.121	0.062	0.803
ACE	136 [99; 173]	111 [92; 135]	98 [90; 127]	114 [89; 131]	0.070	0.333	0.014	0.164	0.109	0.899

* *p*-values are significant at *p* < 0.05; ** data are expressed as median [25% Quartile; 75% Quartile]. Abbreviations: PCOS—polycystic ovary syndrome; ASV—amplicon sequence variant, ACE—abundance-based coverage estimator.

## Data Availability

The primary data on amplicon metasequencing were deposited in the international database NCBI SRA (data: PRJNA899143).

## Supplementary Materials

The following supporting information can be downloaded at: https://www.mdpi.com/article/10.3390/life13010007/s1, Table S1: List of gut microbiome researches on PCOS patients with findings on alpha diversity correlations, Table S2: Sociodemographic characteristics of study participants, Table S3: Short statistics of ASV numbers and alpha diversity indexes representing the gut microbial communities’ richness and diversity. Table S4: Alpha diversity indexes calculated for subgroups of patients with a median relative abundance.

## Abbreviations

17-OHP	17-hydroxyprogesterone
ACE	Abundance-based Coverage Estimator
AFC	Antral Follicle Count
AMH	Anti-Mullerian Hormone
ASV	Amplicon sequence variant
BMI	Body mass index
DHEAS	Dehydroepiandrosterone
DOGMA	Dysbiosis of gut microbiota
ESPEP	Eastern Siberia PCOS Epidemiology & Phenotype
FAI	Free Androgen Index
FSH	Follicle-Stimulating Hormone
HA	Hyperandrogenism
HRT	Hormone therapy
IFG	Impaired fasting glycaemia
IGT	Impaired glucose tolerance
LH	Luteinizing Hormone
LNG-IUD	Levonorgestrel intrauterine device
mF-G	modified Ferriman–Gallwey score for hirsutism
NC-CAH	Nonclassical congenital adrenal hyperplasia
OCP	Oral contraceptive pills
OUT	Operational Taxonomic Unit
PCOS	Polycystic ovary syndrome
PCOM	Polycystic ovarian morphology
REDCap	Research Electronic Data Capture
rRNA	ribosomal RNA
SHBG	Sex-Hormone-Binding Globulin
TSH	Thyroid-Stimulating Hormone
TT	Total Testosterone
U/S	Ultrasound
UNL	Upper Normal Limits
WC	Waist Circumference

## References

[1] Belenkaia L.V., Lazareva L.M., Walker W., Lizneva D.V., Suturina L.V. (2019). Criteria, phenotypes and prevalence of polycystic ovary syndrome. Minerva Ginecol..

[2] Suturina L., Kovacs G.T., Fauser B., Legro R.S. (2022). The Epidemiology of Polycystic Ovary Syndrome. Polycystic Ovary Syndrome.

[3] Cho I., Blaser M.J. (2012). The human microbiome: At the interface of health and disease. Nat. Rev. Genet..

[4] Kho Z.Y., Lal S.K. (2018). The Human Gut Microbiome—A Potential Controller of Wellness and Disease. Front. Microbiol..

[5] Armour C.R., Nayfach S., Pollard K.S., Sharpton T.J. (2019). A Metagenomic Meta-analysis Reveals Functional Signatures of Health and Disease in the Human Gut Microbiome. mSystems.

[6] Tremellen K., Pearce K. (2012). Dysbiosis of gut microbiota (DOGMA)—A novel theory for the development of polycystic ovarian syndrome. Med. Hypotheses.

[7] Lindheim L., Bashir M., Münzker J., Trummer C., Zachhuber V., Leber B., Horvath A., Pieber T.R., Gorkiewicz G., Stadlbauer V. (2017). Alterations in Gut Microbiome Composition and Barrier Function Are Associated with Reproductive and Metabolic Defects in Women with Polycystic Ovary Syndrome (PCOS): A Pilot Study. PLoS ONE.

[8] Liu R., Zhang C., Shi Y., Zhang F., Li L., Wang X., Ling Y., Fu H., Dong W., Shen J. (2017). Dysbiosis of Gut Microbiota Associated with Clinical Parameters in Polycystic Ovary Syndrome. Front. Microbiol..

[9] Insenser M., Murri M., Del Campo R., Ángeles Martínez-García M., Fernández-Durán E., Escobar-Morreale H.F. (2018). Gut Microbiota and the Polycystic Ovary Syndrome: Influence of Sex, Sex Hormones, and Obesity. J. Clin. Endocrinol. Metab..

[10] Torres P.J., Siakowska M., Banaszewska B., Pawelczyk L., Duleba A.J., Kelley S.T., Thackray V.G. (2018). Gut Microbial Diversity in Women With Polycystic Ovary Syndrome Correlates With Hyperandrogenism. J. Clin. Endocrinol. Metab..

[11] Qi X., Yun C., Sun L., Xia J., Wu Q., Wang L., Wang L., Zhang Y., Liang X., Wang L. (2019). Gut microbiota–bile acid–interleukin-22 axis orchestrates polycystic ovary syndrome. Nat. Med..

[12] Zeng B., Lai Z., Sun L., Zhang Z., Yang J., Li Z., Lin J., Zhang Z. (2019). Structural and functional profiles of the gut microbial community in polycystic ovary syndrome with insulin resistance (IR-PCOS): A pilot study. Res. Microbiol..

[13] Zhang J., Sun Z., Jiang S., Bai X., Ma C., Peng Q., Chen K., Chang H., Fang T., Zhang H. (2019). Probiotic Bifidobacterium lactis V9 Regulates the Secretion of Sex Hormones in Polycystic Ovary Syndrome Patients through the Gut-Brain Axis. mSystems.

[14] Chu W., Han Q., Xu J., Wang J., Sun Y., Li W., Chen Z.J., Du Y. (2020). Metagenomic analysis identified microbiome alterations and pathological association between intestinal microbiota and polycystic ovary syndrome. Fertil. Steril..

[15] Eyupoglu N.D., Ergunay K., Acikgoz A., Akyon Y., Yilmaz E., Yildiz B.O. (2020). Gut Microbiota and Oral Contraceptive Use in Overweight and Obese Patients with Polycystic Ovary Syndrome. J. Clin. Endocrinol. Metab..

[16] Haudum C., Lindheim L., Ascani A., Trummer C., Horvath A., Münzker J., Obermayer-Pietsch B. (2020). Impact of Short-Term Isoflavone Intervention in Polycystic Ovary Syndrome (PCOS) Patients on Microbiota Composition and Metagenomics. Nutrients.

[17] Jobira B., Frank D.N., Pyle L., Silveira L.J., Kelsey M.M., Garcia-Reyes Y., Robertson C.E., Ir D., Nadeau K.J., Cree-Green M. (2020). Obese Adolescents With PCOS Have Altered Biodiversity and Relative Abundance in Gastrointestinal Microbiota. J. Clin. Endocrinol. Metab..

[18] Liang Y., Ming Q., Liang J., Zhang Y., Zhang H., Shen T. (2020). Gut microbiota dysbiosis in polycystic ovary syndrome: Association with obesity—A preliminary report. Can. J. Physiol. Pharmacol..

[19] Zhou L., Ni Z., Yu J., Cheng W., Cai Z., Yu C. (2020). Correlation Between Fecal Metabolomics and Gut Microbiota in Obesity and Polycystic Ovary Syndrome. Front. Endocrinol..

[20] Zhou L., Ni Z., Cheng W., Yu J., Sun S., Zhai D., Yu C., Cai Z. (2020). Characteristic gut microbiota and predicted metabolic functions in women with PCOS. Endocr. Connect..

[21] Dong S., Jiao J., Jia S., Li G., Zhang W., Yang K., Wang Z., Liu C., Li D., Wang X. (2021). 16S rDNA Full-Length Assembly Sequencing Technology Analysis of Intestinal Microbiome in Polycystic Ovary Syndrome. Front. Cell. Infect. Microbiol..

[22] Garcia-Beltran C., Malpique R., Carbonetto B., González-Torres P., Henares D., Brotons P., Muñoz-Almagro C., López-Bermejo A., de Zegher F., Ibáñez L. (2021). Gut microbiota in adolescent girls with polycystic ovary syndrome: Effects of randomized treatments. Pediatr. Obes..

[23] He F., Li Y. (2021). The gut microbial composition in polycystic ovary syndrome with insulin resistance: Findings from a normal-weight population. J. Ovarian. Res..

[24] Jobira B., Frank D.N., Silveira L.J., Pyle L., Kelsey M.M., Garcia-Reyes Y., Robertson C.E., Ir D., Nadeau K.J., Cree-Green M. (2021). Hepatic steatosis relates to gastrointestinal microbiota changes in obese girls with polycystic ovary syndrome. PLoS ONE.

[25] Liang Z., Di N., Li L., Yang D. (2021). Gut microbiota alterations reveal potential gut-brain axis changes in polycystic ovary syndrome. J. Endocrinol. Investig..

[26] Mammadova G., Ozkul C., Yilmaz Isikhan S., Acikgoz A., Yildiz B.O. (2021). Characterization of gut microbiota in polycystic ovary syndrome: Findings from a lean population. Eur. J. Clin. Investig..

[27] Ni Z., Cheng W., Ding J., Yao R., Zhang D., Zhai D., Zhou L., Yu C. (2021). Impact of Buzhong Yiqi Prescription on the Gut Microbiota of Patients with Obesity Manifesting Polycystic Ovarian Syndrome. Evid. Based Complement. Alternat. Med..

[28] Zhu X., Li Y., Jiang Y., Zhang J., Duan R., Liu L., Liu C., Xu X., Yu L., Wang Q. (2021). Prediction of Gut Microbial Community Structure and Function in Polycystic Ovary Syndrome With High Low-Density Lipoprotein Cholesterol. Front. Cell. Infect. Microbiol..

[29] Hassan S., Kaakinen M.A., Draisma H., Zudina L., Ganie M.A., Rashid A., Balkhiyarova Z., Kiran G.S., Vogazianos P., Shammas C. (2022). Bifidobacterium Is Enriched in Gut Microbiome of Kashmiri Women with Polycystic Ovary Syndrome. Genes.

[30] Li G., Liu Z., Ren F., Shi H., Zhao Q., Song Y., Fan X., Ma X., Qin G. (2022). Alterations of Gut Microbiome and Fecal Fatty Acids in Patients With Polycystic Ovary Syndrome in Central China. Front. Microbiol..

[31] Tayachew B., Vanden Brink H., Garcia-Reyes Y., Rahat H., D’Alessandro A., Frank D.N., Robertson C.E., Silveira L., Kelsey M., Pyle L. (2022). Combined Oral Contraceptive Treatment Does Not Alter the Gut Microbiome but Affects Amino Acid Metabolism in Sera of Obese Girls With Polycystic Ovary Syndrome. Front. Physiol..

[32] Wang X., Xu T., Liu R., Wu G., Gu L., Zhang Y., Zhang F., Fu H., Ling Y., Wei X. (2022). High-Fiber Diet or Combined With Acarbose Alleviates Heterogeneous Phenotypes of Polycystic Ovary Syndrome by Regulating Gut Microbiota. Front. Endocrinol..

[33] Lüll K., Arffman R.K., Sola-Leyva A., Molina N.M., Aasmets O., Herzig K.H., Plaza-Díaz J., Franks S., Morin-Papunen L., Tapanainen J.S. (2021). The Gut Microbiome in Polycystic Ovary Syndrome and Its Association with Metabolic Traits. J. Clin. Endocrinol. Metab..

[34] Schloss P.D., Handelsman J. (2005). Introducing DOTUR, a computer program for defining operational taxonomic units and estimating species richness. Appl. Environ. Microbiol..

[35] Schloss P.D., Westcott S.L., Ryabin T., Hall J.R., Hartmann M., Hollister E.B., Lesniewski R.A., Oakley B.B., Parks D.H., Robinson C.J. (2009). Introducing mothur: Open-source, platform-independent, community-supported software for describing and comparing microbial communities. Appl. Environ. Microbiol..

[36] Chao A., Chazdon R.L., Colwell R.K., Shen T.J. (2006). Abundance-based similarity indices and their estimation hen there are unseen species in samples. Biometrics.

[37] Kim B.R., Shin J., Guevarra R., Lee J.H., Kim D.W., Seol K.H., Lee J.H., Kim H.B., Isaacson R. (2017). Deciphering Diversity Indices for a Better Understanding of Microbial Communities. J. Microbiol. Biotechnol..

[38] Hughes J.B., Hellmann J.J., Ricketts T.H., Bohannan B.J. (2001). Counting the uncountable: Statistical approaches to estimating microbial diversity. Appl. Environ. Microbiol..

[39] Teede H.J., Misso M.L., Costello M.F., Dokras A., Laven J., Moran L., Piltonen T., Norman R.J., International PCOS Network (2018). Recommendations from the international evidence-based guideline for the assessment and management of polycystic ovary syndrome. Fertil. Steril..

[40] Yildiz B.O., Bolour S., Woods K., Moore A., Azziz R. (2010). Visually scoring hirsutism. Hum. Reprod. Update.

[41] Bolyen E., Rideout J.R., Dillon M.R., Bokulich N.A., Abnet C.C., Al-Ghalith G.A., Alexander H., Alm E.J., Arumugam M., Asnicar F. (2019). Reproducible, interactive, scalable and extensible microbiome data science using QIIME 2. Nat. Biotechnol..

[42] Naing L., Winn T., Rusli B.N. (2006). Practical issues in calculating the sample size for prevalence studies. Med. Stat..

[43] Atalyan A.V., Kolesnikova L.I., Kolesnikov S.I., Grjibovski A.M., Suturina L.V. (2019). Research Electronic Data Capture (REDCap) for Building and Managing Databases for Population-based Biomedical Studies. Ekol. Cheloveka (Hum. Ecol.).

[44] Zuur A.F., Ieno E.N., Elphick C.S. (2010). A protocol for data exploration to avoid common statistical problems. Methods Ecol. Evol..

[45] Lehmann R. (2013). 3sigma-Rule for Outlier Detection from the Viewpoint of Geodetic Adjustment. J. Surv. Eng..

[46] Martínez I., Muller C.E., Walter J. (2013). Long-term temporal analysis of the human fecal microbiota revealed a stable core of dominant bacterial species. PLoS ONE.

[47] Costa M., Weese J.S. (2019). Methods and basic concepts for microbiota assessment. Vet. J..

[48] Lindheim L., Bashir M., Münzker J., Trummer C., Zachhuber V., Pieber T.R., Gorkiewicz G., Obermayer-Pietsch B. (2016). The Salivary Microbiome in Polycystic Ovary Syndrome (PCOS) and Its Association with Disease-Related Parameters: A Pilot Study. Front. Microbiol..

[49] Li N., Li Y., Qian C., Liu Q., Cao W., Ma M., He R., Chen R., Geng R., Liu Y. (2021). Dysbiosis of the Saliva Microbiome in Patients With Polycystic Ovary Syndrome. Front. Cell. Infect. Microbiol..

[50] Hong X., Qin P., Huang K., Ding X., Ma J., Xuan Y., Zhu X., Peng D., Wang B. (2020). Association between polycystic ovary syndrome and the vaginal microbiome: A case-control study. Clin. Endocrinol..

[51] Tu Y., Zheng G., Ding G., Wu Y., Xi J., Ge Y., Gu H., Wang Y., Sheng J., Liu X. (2020). Comparative Analysis of Lower Genital Tract Microbiome Between PCOS and Healthy Women. Front. Physiol..

[52] Lu C., Wang H., Yang J., Zhang X., Chen Y., Feng R., Qian Y. (2021). Changes in Vaginal Microbiome Diversity in Women With Polycystic Ovary Syndrome. Front. Cell. Infect. Microbiol..

[53] Wang Q., Wang Q., Zhao L., Bin Y., Wang L., Wang L., Zhang K., Li Q. (2022). Blood Bacterial 16S rRNA Gene Alterations in Women With Polycystic Ovary Syndrome. Front. Endocrinol..

[54] Huang L., Wu X., Guo S., Lv Y., Zhou P., Huang G., Duan Z., Sun W. (2022). Metagenomic-based characterization of the gut virome in patients with polycystic ovary syndrome. Front. Microbiol..

