# Supplementary material for: Polycystic Ovary Syndrome and Gut Microbiota: Phenotype Matters
Source: Life (Basel). 2022 Dec 20;13(1):7. doi: 10.3390/life13010007 (PMC9861125; doi:10.3390/life13010007)
Supplement: Supplementary file 1 [file life-13-00007-s001.zip › Table S1.pdf]

Table S1. List of gut microbiome researches in PCOS patients with findings alpha diversity correlations

	Reference	Country	Participants	Studied indexes	Sequencing method	Alpha diversity indexes		
						Observed up in PCOS	Observed down in PCOS	No difference in PCOS
[7]	Lindheim et al., 2017	Austria	PCOS, n = 24<br>Control, n = 19	OTUs, Faith phylogenetic diversity	Amplicon sequencing (V1-V2 regions)		Faith's phylogenetic diversity<br>observed OTUs	
[8]	Liu et al., 2017	China	PCOS, n = 33: obese PCOS, n = 21; non-obese PCOS, n = 12<br>Control, n = 15: obese control, n = 6; non-obese control, n = 9	OTUs, Shannon, Simpson, Chao	Amplicon sequencing (V3-V4 regions)		OTUs<br>Chao	Shannon<br>Simpson
[9]	Insenser et al., 2018	Spain	PCOS, n = 15: obese PCOS, n = 8; non-obese PCOS, n = 7<br>Control, n = 16: obese control, n = 8; non-obese control, n = 8<br>Healthy men, n = 15: Obese men, n = 7; Non-obese men, n = 8	OTUs, Shannon, Chao, Jaccard, Sorensen	Amplicon sequencing (V4 region)	Shannon	Chao	Jaccard, Sorensen
[10]	Torres et al., 2018	Poland	PCOS, n = 73<br>PCOM, n = 42<br>Control, n = 48	observed SVs, Faith PD, Shannon, Pielou	Amplicon sequencing (V4 regions)		observed SVs<br>Faith PD<br>Shannon<br>evenness of SVs	
[11]	Qi et al., 2019	China	PCOS, n = 50<br>Control, n = 43	Shannon	Amplicon sequencing (V3-V4 regions)<br>Whole metagenome shotgun sequencing			Shannon
[12]	Zeng et al., 2019	China	PCOS, n = 17: IR-PCOS, n = 9; Non-IR-PCOS, n = 8<br>Control, n = 8	observed OTUs, Shannon	Amplicon sequencing (V3-V4 regions)		observed OTUs<br>Shannon	
[13]	Zhang et al., 2019	China	PCOS, n = 38<br>Control, n = 26 [1]	Not used	Amplicon sequencing (V3-V4 regions)			
[14]	Chu et al., 2020	China	PCOS, n = 14<br>Control, n = 14	Not used	Whole metagenome shotgun sequencing			

[15]	Eyupoglu et al., 2020	Turkey	Obese PCOS, n = 17<br>Obese Control, n = 15	OTUs, Faith PD, Pielou, Jaccard, Shannon	Amplicon sequencing (V3-V4 regions)		Observed OTUs	Faith PD<br>Pielou<br>Shannon
[16]	Haudum et al., 2020	Austria	PCOS, n = 24<br>Control, n = 20 [2]	OTUs, Faith PD, Shannon	Amplicon sequencing (V1-V2 regions)		Shannon<br>Faith PD	
[17]	Jobira et al., 2020	USA	PCOS, n = 37<br>Control, n = 21 [3]	OTUs, Shannon diversity, Shannon evenness, richness (Sobs)	Amplicon sequencing (V3-V4 regions)		OTU, Shannon diversity, Shannon evenness	richness (Sobs)
[18]	Liang et al., 2020	China	PCOS, n = 27: obese PCOS, n = 8; non-obese PCOS, n = 10<br>Control, n = 9	OTUs, Chao, Shannon	Amplicon sequencing (V3-V4 regions)		observed SVs<br>Chao<br>Shannon	
[19]	Zhou et al., 2020a	China	PCOS, n = 60: obese PCOS, n = 30; non-obese PCOS, n = 30<br>Control, n = 41: obese control, n = 11; non-obese control, n = 30	OTUs, Sobs, Chao, ACE, Simpson, Shannon	Amplicon sequencing (V3-V4 regions)	Simpson	Sobs<br>Chao<br>ACE	Shannon
[20]	Zhou et al., 2020b	China	PCOS with obesity, n = 18<br>Control with obesity, n = 15	OTUs, Shannon, Sobs, ACE	Amplicon sequencing (V1-V2 regions)		Sobs<br>Shannon	ACE
[21]	Dong et al., 2021	China	PCOS, n = 45: obese PCOS, n = 31; non-obese PCOS, n = 14<br>Control, n = 37: obese control, n = 12; non-obese control, n = 25	Shannon	16S rDNA full-length assembly meta-sequencing		Shannon	
[22]	Garcia-Beltran et al., 2021	Spain	PCOS, n = 23<br>Control, n = 31 [3, 4]	ASVs, Pielou's Evenness, Shannon	Amplicon sequencing (V3-V4 regions)		Pielou's Evenness<br>Shannon's	ASVs
[23]	He et al., 2021	China	non-obese PCOS, n = 26: NIR-PCOS, n = 10; IR-PCOS, n = 14<br>Control, n = 12	Chao, Observed OTUs	Amplicon sequencing (V3-V4 regions)		observed OTUs<br>Chao	
[24]	Jobira et al., 2021	USA	PCOS, n = 34: PCOS with Hepatic Steatosis, n = 17;<br>PCOS without Hepatic Steatosis, n = 17	OTUs, Shannon diversity, Shannon evenness, richness (Sobs)	Amplicon sequencing (V3-V4 regions)	Shannon diversity	Shannon evenness<br>richness (Sobs)	

[25]	Liang et al., 2021	China	PCOS, n = 20: Lean PCOS, n = 10; Overweight PCOS, n = 10<br>Control, n = 20: Lean control, n = 10; Overweight control, n = 10	OTUs, Sobs, Chao, ACE, Shannon	Amplicon sequencing (V4 region)		observed OTUs<br>Sobs<br>Chao<br>Ace<br>Shannon	
[26]	Mammadova et al., 2021	Turkey	PCOS, phenotype A, n = 24<br>Control, n = 22	OTUs, Shannon, Chao	Amplicon sequencing (V3-V4 regions)			Shannon<br>Simpson<br>Chao<br>Faith's PD<br>observed OTUs
[27]	Ni et al., 2021	China	Obese PCOS with spleen deficiency, n = 15 [5]	Sobs, Chao, Shannon, Simpson	Amplicon sequencing (V3-V4 regions)			Sobs<br>Chao<br>Shannon<br>Simpson
[28]	Zhu et al., 2021	China	PCOS, n = 54: high LDLC PCOS, n = 16; low LDLC PCOS, n = 38<br>Control, n = 33: high LDLC Control, n = 15; low LDLC Control, n = Control, n = 18	OTUs, Shannon	Amplicon sequencing (V3-V4 regions)		Shannon	
[29]	Hassan et al., 2022	India	PCOS, n = 20<br>Control, n = 20 [6]	OTUs, Shannon	Amplicon sequencing (V3-V4 regions)	Shannon		
[30]	Li et al., 2022	China	PCOS, n = 31: PCOSF group, N = 12; PCOST group, N = 19<br>Control, n = 27	ACE, Chao, Shannon, Simpson	Amplicon sequencing (V3-V4 regions)			ACE<br>Chao<br>Shannon<br>Simpson
[31]	Tayachew et al., 2022	USA	Obese PCOS, n = 29:<br>case – oral contraceptive use, n = 8; control – no treatment for PCOS, n = 21 [3]	Shannon diversity, Shannon evenness, richness (Sobs)	Amplicon sequencing (V3-V4 regions)			Shannon diversity<br>Shannon evenness<br>richness (Sobs)
[32]	Wang et al., 2022a	China	PCOS, n = 25: W group – PCOS with WTP diet, n = 14; A group –	observed ASVs, Shannon	Amplicon sequencing (V3-V4 regions)		observed ASVs<br>Shannon	

[33]	Lüll et al., 2021	Finland	PCOS with WTP diet and acarbose, n = 11<br>PCOS, n = 102;<br>non-PCOS control, n = 202	ASV, Shannon, Inverse Simpson	Amplicon sequencing (V3-V4 regions)	ASV, Shannon, Inverse Simpson

Additional information on an interventionary experimental protocol or on research with adolescent girls and college and school students:

[1] this study is a two phase experiment in which in the first stage, an imbalance in the intestinal microbiota of the patients with PCOS was studied; whereas on the second stage, the impact of the probiotic *Bifidobacterium lactis* V9 on the intestinal microbiome, gut-brain mediators, and sex hormones of 14 patients with PCOS was monitored;

[2] this study aimed to explore clinical and biochemical parameters, urine isoflavone levels in PCOS and control women before and three days after a defined isoflavone intervention via soy milk;

[3] Adolescent girls;

[4] this study hypothesized that the composition of the gut microbiota in adolescent girls with PCOS and without obesity will differ from that in control girls and will have a dissimilar pattern after a low-dose combination of one mixed anti-androgen and antimineralocorticoid (spironolactone), and two insulin sensitizers (pioglitazone and metformin) or oral contraceptive administration;

[5] this study aimed to explore gut microbiota and fecal metabolite alterations in such patients treated with BZYQ (Buzhong Yiqi);

[6] Women were recruited during regional PCOS screening of college and school students in different schools and colleges in Kashmir, North India.

Abbreviation:

PCOS – polycystic ovary syndrome; OTU – Operational Taxonomic Unit; PCOM – polycystic ovarian morphology; SV – sequence variants; Faith PD - Faith phylogenetic diversity; ACE – Abundance-based Coverage Estimator; IR – insulin resistance; ASV – Amplicon sequence variant; BZYQ – Buzhong Yiqi; LDLC – low-density lipoprotein cholesterol; WTP diet – a high-fiber diet, traditional Chinese medicinal foods, and prebiotics.
