# Supplementary material for: Polycystic Ovary Syndrome and Gut Microbiota: Phenotype Matters
Source: Life (Basel). 2022 Dec 20;13(1):7. doi: 10.3390/life13010007 (PMC9861125; doi:10.3390/life13010007)
Supplement: Supplementary file 1 [file life-13-00007-s001.zip › Table S2.pdf]

Table S2. Sociodemographic characteristics of study participants.

Parameter	n=184
Age. years. M±SD	33.18±6.14
Race. n/N (%)	
Caucasians	133/184 (72.28)
Asians	31/184 (16.85)
Mixed race	20/184 (10.87)
Place of residence. n/N (%)	
city	108/184 (58.70)
village	76/184 (41.30)
Marital status. n/N (%)	
Single	55/184 (29.89)
Married	87/184 (47.28)
Living with another	24/184 (13.04)
Separated	1/184 (0.54)
Divorced	15/184 (8.15)
Widowed	2/184 (1.09)
Would rather not say	0/184 (0.00)
Occupation. n/N (%)	
Legislators senior officials and managers	4/184 (2.17)
Professionals	81/184 (44.02)
Technicians and associate professionals	31/184 (16.85)
Office clerks	31/184 (16.85)
Service workers and shop and market sales	14/184 (7.61)
Skilled agricultural and fishery workers	2/184 (1.09)
Craft and related trades workers	9/184 (4.89)
Plant and machine operators and assemblers	3/184 (1.63)
Elementary occupations	7/184 (3.80)
Armed forces	2/184 (1.09)
Education. n/N (%)	
Doctoral degree	16/184 (8.70)
Master's degree	123/184 (66.85)
Incomplete High school	8/184 (4.35)
Bachelor's degree	30/184 (16.30)
Some college	0/184 (0.00)
High school equivalent	5/184 (2.72)
Middle school	1/184 (0.54)
No degree	1/184 (0.54)
