# Supplementary material for: Polycystic Ovary Syndrome and Gut Microbiota: Phenotype Matters
Source: Life (Basel). 2022 Dec 20;13(1):7. doi: 10.3390/life13010007 (PMC9861125; doi:10.3390/life13010007)
Supplement: Supplementary file 1 [file life-13-00007-s001.zip › Table S4.pdf]

Table S4. Alpha diversity indexes calculated for subgroups of patients with a median relative abundance

Indexes	Non-PCOS n=121		PCOS n=63		p-value*					
	Control (1a) n=19	"Grey zone" (1b) n=102	Phenotypes A,B,C (2a) n=41	Phenotype D (2b) n=22	1a-1b	2a-2b	1a-2a	1a-2b	1b-2a	1b-2b
ASV	131** [85; 166]	113 [91;137]	101 [84; 130]	116 [93; 131]	0.140	0.108	**0.045**	0.248	0.127	0.823
Shannon	5.96 [5.36; 6.54]	5.53 [4.91;6.04]	5.23 [4.80; 5.84]	5.41 [4.76;5.79]	**0.016**	0.880	**0.003**	**0.014**	0.203	0.357
Simpson	0.97 [0.94; 0.98]	0.95 [0.92;0.97]	0.94 [0.92; 0.97]	0.94 [0.91; 0.97]	**0.017**	0.971	**0.006**	**0.017**	0.351	0.472
Chao	139 [111; 192]	116 [99;151]	108 [91; 130]	117 [96; 148]	0.075	0.285	**0.010**	0.121	0.062	0.803
ACE	136 [99; 173]	111 [92;135]	98 [90; 127]	114 [89; 131]	0.070	0.333	**0.014**	0.164	0.109	0.899

* – p-values are significant at $p < 0.05$;

** – data are expressed as median [25% Quartile; 75% Quartile].

Abbreviations: PCOS – polycystic ovary syndrome; ASV – Amplicon sequence variant, ACE – Abundance-based Coverage Estimator.
